# OLDER ADULT ANIMAL FARMERS’ EVACUATION EFFORTS DURING THE 2021 PACIFIC NORTHWEST FLOODS IN CANADA

**DOI:** 10.1093/geroni/igae098.1708

**Published:** 2024-12-31

**Authors:** Haorui Wu

**Affiliations:** Dalhousie University, Halifax, Nova Scotia, Canada

## Abstract

The animal farming communities in the Fraser Valley were devastated by the 2021 Pacific Northwest floods. The community residents, community-based service and professional agencies, and government emergency responders were engaged in the evacuation. Through qualitative interviews of animal farm owners, community-based service agencies, and governmental emergency responders, this project discovers that during the decision-making stage of the disaster evacuation, the older adult animal farmers’ their long-term engagement with their natural, built, and social environments enabled them to develop the initiatives for evacuation strategies help the governmental officials better allocate resources and support community-based service and professional organizations to coordinate different community-based services and resources to improve the evacuation process. This collaboration shed light on an innovative approach for leverage older adult animal farmers’ expertise to facilitate effective emergency decision-making.

